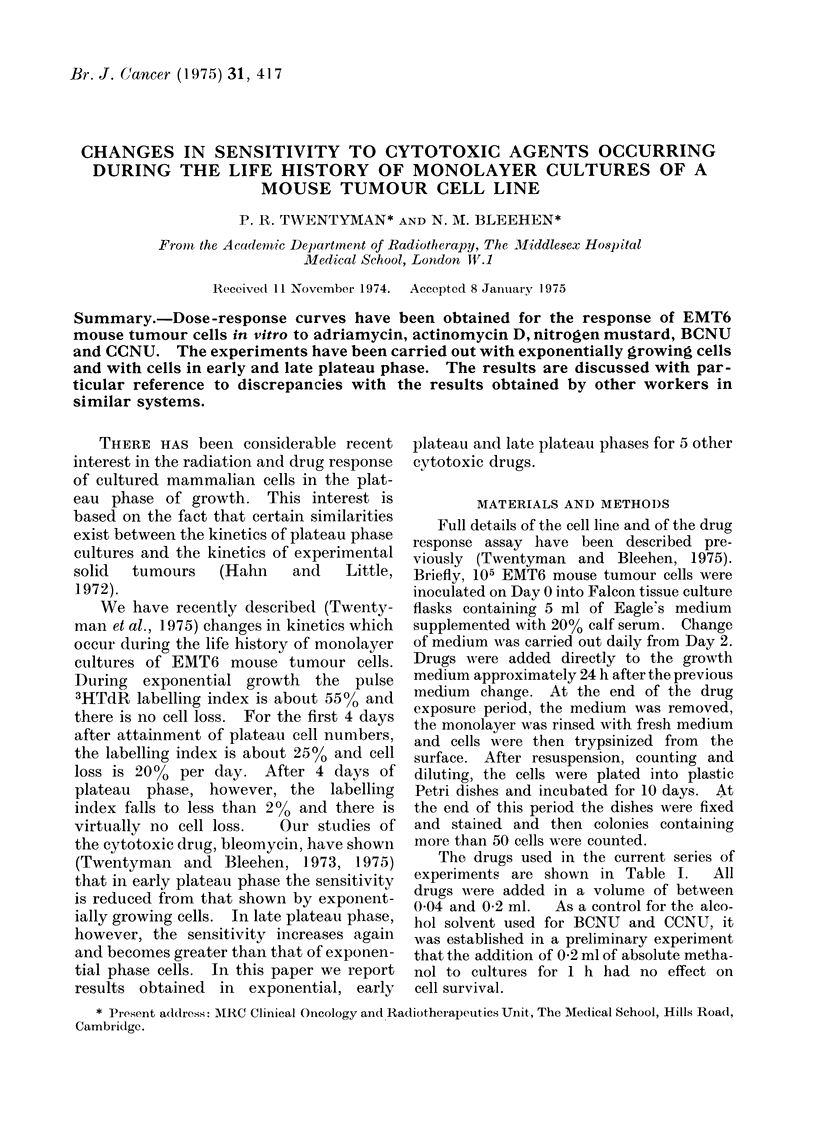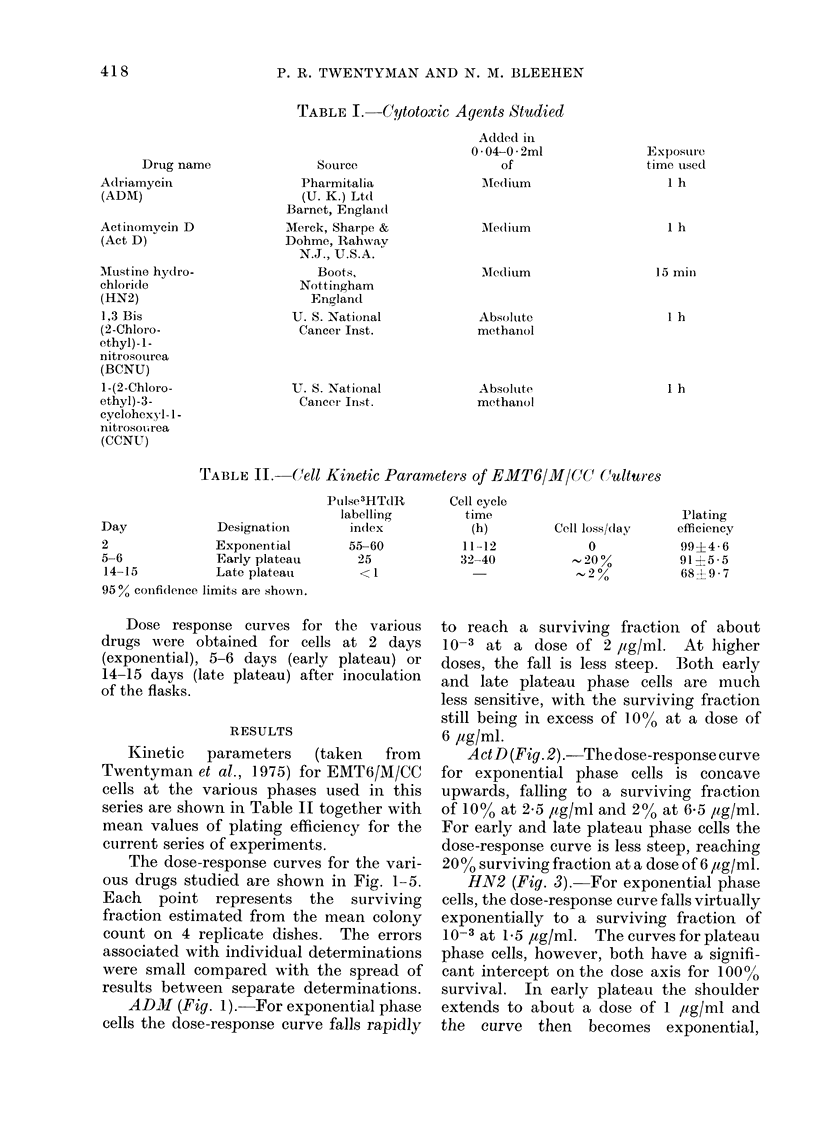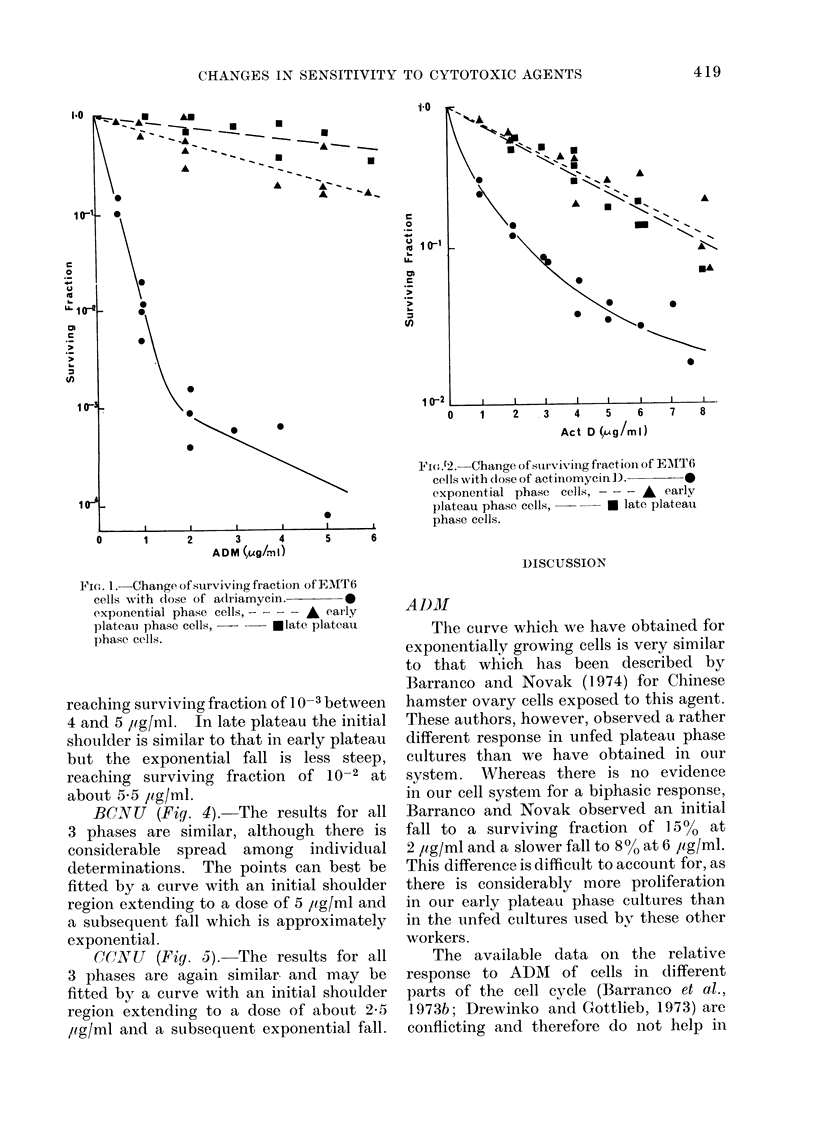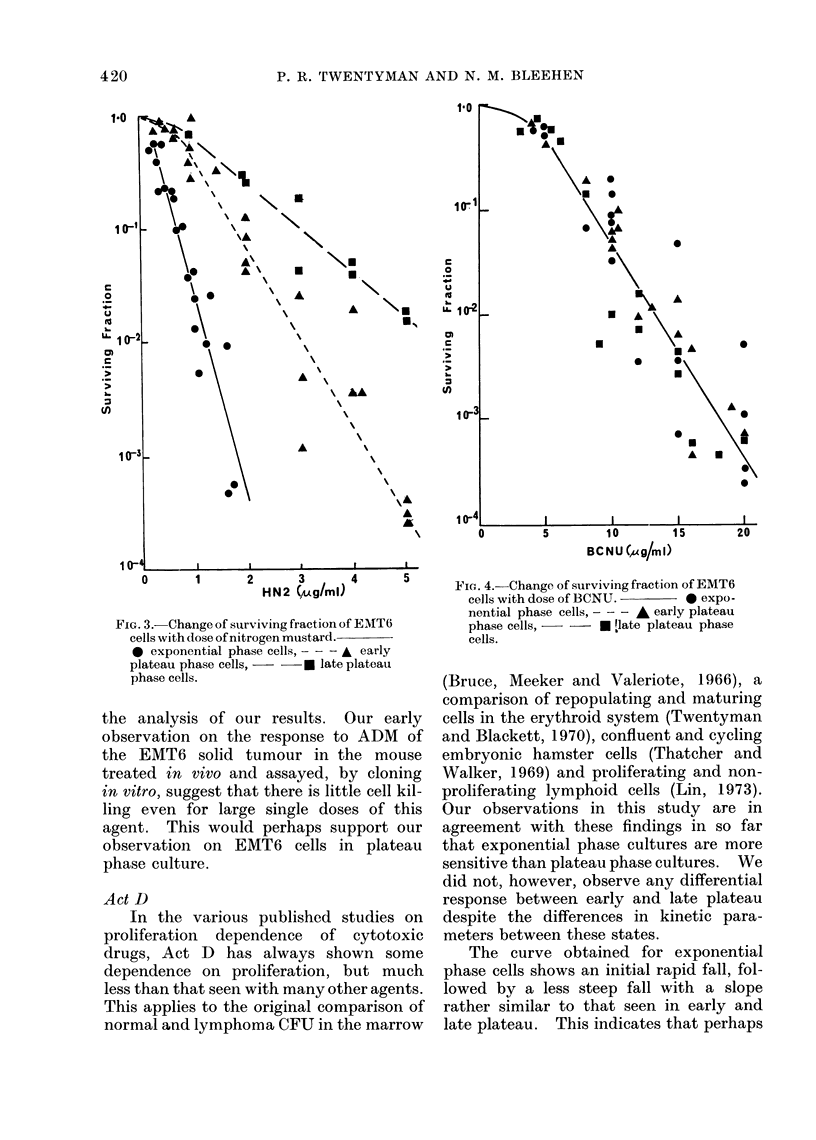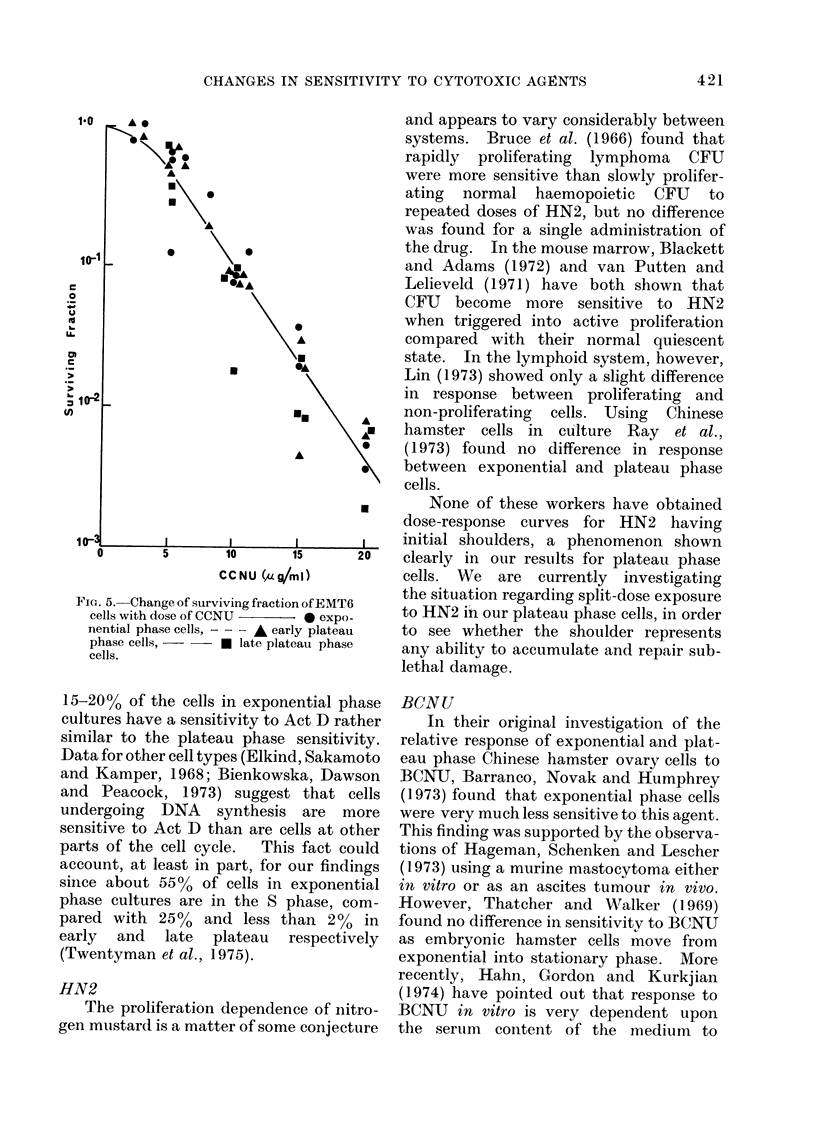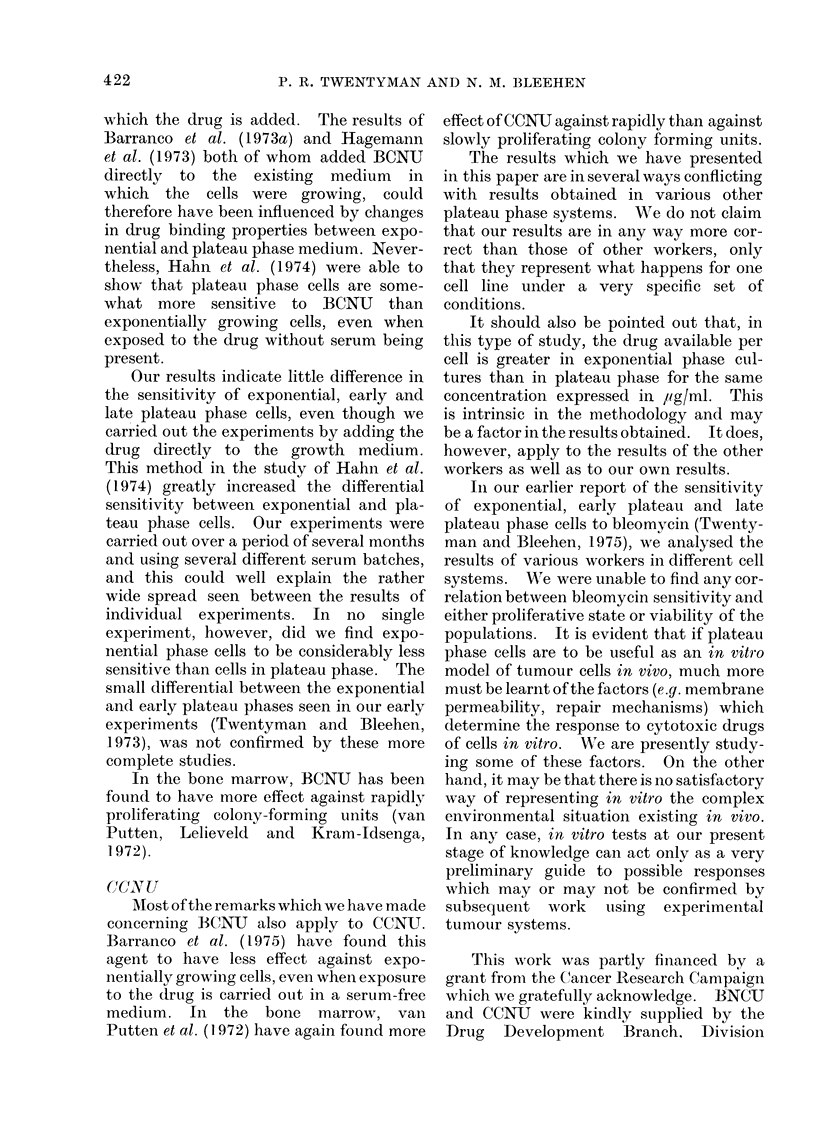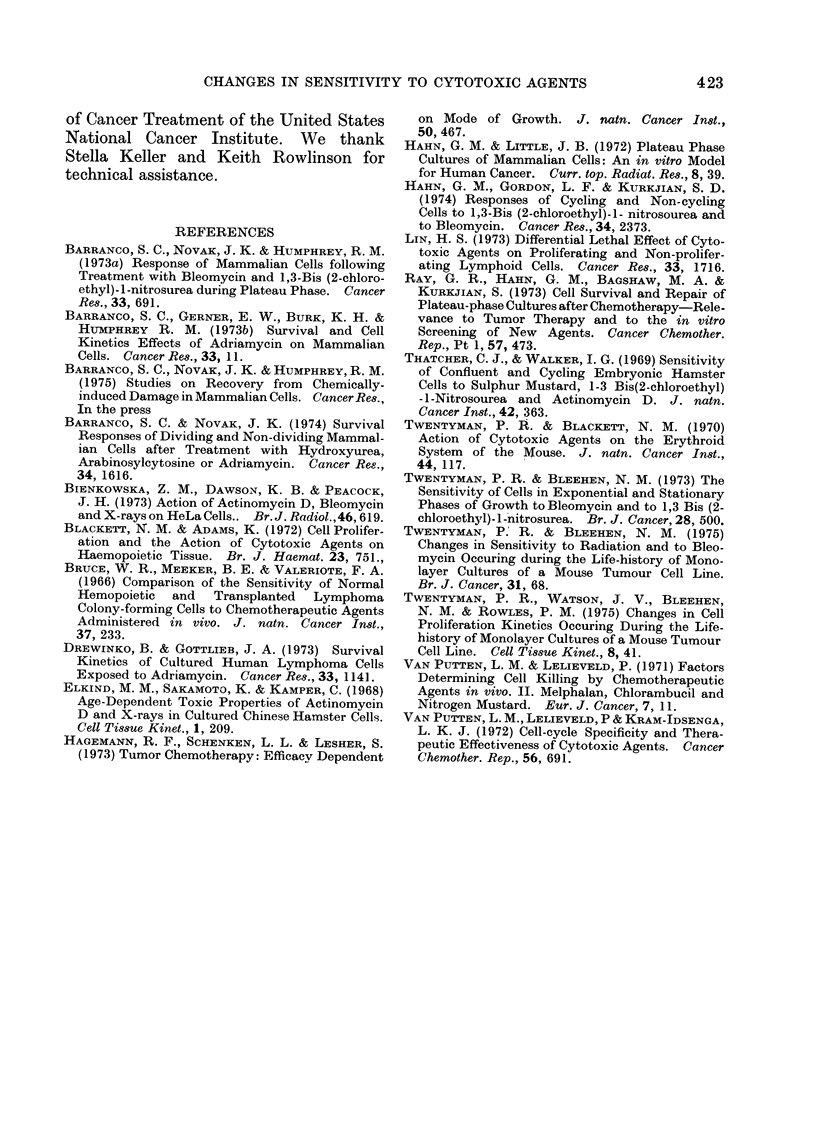# Changes in sensitivity to cytotoxic agents occurring during the life history of monolayer cultures of a mouse tumour cell line.

**DOI:** 10.1038/bjc.1975.80

**Published:** 1975-04

**Authors:** P. R. Twentyman, N. M. Bleehen

## Abstract

Dose-response curves have been obtained for the response of EMT6 mouse tumour cells in vitro to adriamycin, actinomycin D, nitrogen mustard, BCNU and CCNU. The experiments have been carried out with exponentially growing cells and with cells in early and late plateau phase. The results are discussed with particular reference to discrepancies with the results obtained by other workers in similar systems.


*Br. J. Cancer* (1975) **31**, 417

### CHANGES IN SENSITIVITY TO CYTOTOXIC AGENTS OCCURRING DURING THE LIFE HISTORY OF MONOLAYER CULTURES OF A MOUSE TUMOUR CELL LINE

P. R. TWENTYMAN* AND N. M. BLEEHEN*

*From the Academic Department of Radiotherapy, The Middlesex Hospital Medical School, London W.1*

Received 11 November 1974. Accepted 8 January 1975

**Summary.—Dose-response curves have been obtained for the response of EMT6 mouse tumour cells *in vitro* to adriamycin, actinomycin D, nitrogen mustard, BCNU and CCNU. The experiments have been carried out with exponentially growing cells and with cells in early and late plateau phase. The results are discussed with particular reference to discrepancies with the results obtained by other workers in similar systems.**

THERE HAS been considerable recent interest in the radiation and drug response of cultured mammalian cells in the plateau phase of growth. This interest is based on the fact that certain similarities exist between the kinetics of plateau phase cultures and the kinetics of experimental solid tumours (Hahn and Little, 1972).

We have recently described (Twentyman *et al.*, 1975) changes in kinetics which occur during the life history of monolayer cultures of EMT6 mouse tumour cells. During exponential growth the pulse $^3$HTdR labelling index is about 55% and there is no cell loss. For the first 4 days after attainment of plateau cell numbers, the labelling index is about 25% and cell loss is 20% per day. After 4 days of plateau phase, however, the labelling index falls to less than 2% and there is virtually no cell loss. Our studies of the cytotoxic drug, bleomycin, have shown (Twentyman and Bleehen, 1973, 1975) that in early plateau phase the sensitivity is reduced from that shown by exponentially growing cells. In late plateau phase, however, the sensitivity increases again and becomes greater than that of exponential phase cells. In this paper we report results obtained in exponential, early plateau and late plateau phases for 5 other cytotoxic drugs.

### MATERIALS AND METHODS

Full details of the cell line and of the drug response assay have been described previously (Twentyman and Bleehen, 1975). Briefly, $10^5$ EMT6 mouse tumour cells were inoculated on Day 0 into Falcon tissue culture flasks containing 5 ml of Eagle's medium supplemented with 20% calf serum. Change of medium was carried out daily from Day 2. Drugs were added directly to the growth medium approximately 24 h after the previous medium change. At the end of the drug exposure period, the medium was removed, the monolayer was rinsed with fresh medium and cells were then trypsinized from the surface. After resuspension, counting and diluting, the cells were plated into plastic Petri dishes and incubated for 10 days. At the end of this period the dishes were fixed and stained and then colonies containing more than 50 cells were counted.

The drugs used in the current series of experiments are shown in Table I. All drugs were added in a volume of between 0·04 and 0·2 ml. As a control for the alcohol solvent used for BCNU and CCNU, it was established in a preliminary experiment that the addition of 0·2 ml of absolute methanol to cultures for 1 h had no effect on cell survival.

* Present address: MRC Clinical Oncology and Radiotherapeutics Unit, The Medical School, Hills Road, Cambridge.

TABLE I.—*Cytotoxic Agents Studied*

| Drug name | Source | Added in 0·04–0·2ml of | Exposure time used |
|---|---|---|---|
| Adriamycin (ADM) | Pharmitalia (U. K.) Ltd Barnet, England | Medium | 1 h |
| Actinomycin D (Act D) | Merck, Sharpe & Dohme, Rahway N.J., U.S.A. | Medium | 1 h |
| Mustine hydrochloride (HN2) | Boots, Nottingham England | Medium | 15 min |
| 1,3 Bis (2-Chloroethyl)-1-nitrosourea (BCNU) | U. S. National Cancer Inst. | Absolute methanol | 1 h |
| 1-(2-Chloroethyl)-3-cyclohexyl-1-nitrosourea (CCNU) | U. S. National Cancer Inst. | Absolute methanol | 1 h |

TABLE II.—*Cell Kinetic Parameters of EMT6/M/CC Cultures*

| Day | Designation | Pulse $^3$HTdR labelling index | Cell cycle time (h) | Cell loss/day | Plating efficiency |
|---|---|---|---|---|---|
| 2 | Exponential | 55–60 | 11–12 | 0 | $99\pm4\cdot6$ |
| 5–6 | Early plateau | 25 | 32–40 | ~20% | $91\pm5\cdot5$ |
| 14–15 | Late plateau | <1 | — | ~2% | $68\pm9\cdot7$ |

95% confidence limits are shown.

Dose response curves for the various drugs were obtained for cells at 2 days (exponential), 5–6 days (early plateau) or 14–15 days (late plateau) after inoculation of the flasks.

### RESULTS

Kinetic parameters (taken from Twentyman *et al.*, 1975) for EMT6/M/CC cells at the various phases used in this series are shown in Table II together with mean values of plating efficiency for the current series of experiments.

The dose-response curves for the various drugs studied are shown in Fig. 1–5. Each point represents the surviving fraction estimated from the mean colony count on 4 replicate dishes. The errors associated with individual determinations were small compared with the spread of results between separate determinations.

*ADM* (*Fig.* 1).—For exponential phase cells the dose-response curve falls rapidly to reach a surviving fraction of about $10^{-3}$ at a dose of 2 μg/ml. At higher doses, the fall is less steep. Both early and late plateau phase cells are much less sensitive, with the surviving fraction still being in excess of 10% at a dose of 6 μg/ml.

*Act D* (*Fig.* 2).—The dose-response curve for exponential phase cells is concave upwards, falling to a surviving fraction of 10% at 2·5 μg/ml and 2% at 6·5 μg/ml. For early and late plateau phase cells the dose-response curve is less steep, reaching 20% surviving fraction at a dose of 6 μg/ml.

*HN2* (*Fig.* 3).—For exponential phase cells, the dose-response curve falls virtually exponentially to a surviving fraction of $10^{-3}$ at 1·5 μg/ml. The curves for plateau phase cells, however, both have a significant intercept on the dose axis for 100% survival. In early plateau the shoulder extends to about a dose of 1 μg/ml and the curve then becomes exponential,

Fig. 1.—Change of surviving fraction of EMT6 cells with dose of adriamycin.———● exponential phase cells, – – – – ▲ early plateau phase cells, —— —— ■late plateau phase cells.

Fig. 2.—Change of surviving fraction of EMT6 cells with dose of actinomycin D.———● exponential phase cells, – – – ▲ early plateau phase cells, —— —— ■ late plateau phase cells.

reaching surviving fraction of $10^{-3}$ between 4 and 5 μg/ml. In late plateau the initial shoulder is similar to that in early plateau but the exponential fall is less steep, reaching surviving fraction of $10^{-2}$ at about 5·5 μg/ml.

*BCNU* (*Fig. 4*).—The results for all 3 phases are similar, although there is considerable spread among individual determinations. The points can best be fitted by a curve with an initial shoulder region extending to a dose of 5 μg/ml and a subsequent fall which is approximately exponential.

*CCNU* (*Fig. 5*).—The results for all 3 phases are again similar and may be fitted by a curve with an initial shoulder region extending to a dose of about 2·5 μg/ml and a subsequent exponential fall.

### DISCUSSION

### *ADM*

The curve which we have obtained for exponentially growing cells is very similar to that which has been described by Barranco and Novak (1974) for Chinese hamster ovary cells exposed to this agent. These authors, however, observed a rather different response in unfed plateau phase cultures than we have obtained in our system. Whereas there is no evidence in our cell system for a biphasic response, Barranco and Novak observed an initial fall to a surviving fraction of 15% at 2 μg/ml and a slower fall to 8% at 6 μg/ml. This difference is difficult to account for, as there is considerably more proliferation in our early plateau phase cultures than in the unfed cultures used by these other workers.

The available data on the relative response to ADM of cells in different parts of the cell cycle (Barranco *et al.*, 1973*b*; Drewinko and Gottlieb, 1973) are conflicting and therefore do not help in

FIG. 3.—Change of surviving fraction of EMT6 cells with dose of nitrogen mustard.——— ● exponential phase cells, – – – ▲ early plateau phase cells, —— ——■ late plateau phase cells.

FIG. 4.—Change of surviving fraction of EMT6 cells with dose of BCNU. ——— ● exponential phase cells, – – – ▲ early plateau phase cells, —— —— ■ late plateau phase cells.

the analysis of our results. Our early observation on the response to ADM of the EMT6 solid tumour in the mouse treated *in vivo* and assayed, by cloning *in vitro*, suggest that there is little cell killing even for large single doses of this agent. This would perhaps support our observation on EMT6 cells in plateau phase culture.

*Act D*

In the various published studies on proliferation dependence of cytotoxic drugs, Act D has always shown some dependence on proliferation, but much less than that seen with many other agents. This applies to the original comparison of normal and lymphoma CFU in the marrow (Bruce, Meeker and Valeriote, 1966), a comparison of repopulating and maturing cells in the erythroid system (Twentyman and Blackett, 1970), confluent and cycling embryonic hamster cells (Thatcher and Walker, 1969) and proliferating and non-proliferating lymphoid cells (Lin, 1973). Our observations in this study are in agreement with these findings in so far that exponential phase cultures are more sensitive than plateau phase cultures. We did not, however, observe any differential response between early and late plateau despite the differences in kinetic parameters between these states.

The curve obtained for exponential phase cells shows an initial rapid fall, followed by a less steep fall with a slope rather similar to that seen in early and late plateau. This indicates that perhaps

FIG. 5.—Change of surviving fraction of EMT6 cells with dose of CCNU ——— ● exponential phase cells, – – – ▲ early plateau phase cells, —— —— ■ late plateau phase cells.

15–20% of the cells in exponential phase cultures have a sensitivity to Act D rather similar to the plateau phase sensitivity. Data for other cell types (Elkind, Sakamoto and Kamper, 1968; Bienkowska, Dawson and Peacock, 1973) suggest that cells undergoing DNA synthesis are more sensitive to Act D than are cells at other parts of the cell cycle. This fact could account, at least in part, for our findings since about 55% of cells in exponential phase cultures are in the S phase, compared with 25% and less than 2% in early and late plateau respectively (Twentyman *et al.*, 1975).

*HN2*

The proliferation dependence of nitrogen mustard is a matter of some conjecture and appears to vary considerably between systems. Bruce *et al.* (1966) found that rapidly proliferating lymphoma CFU were more sensitive than slowly proliferating normal haemopoietic CFU to repeated doses of HN2, but no difference was found for a single administration of the drug. In the mouse marrow, Blackett and Adams (1972) and van Putten and Lelieveld (1971) have both shown that CFU become more sensitive to HN2 when triggered into active proliferation compared with their normal quiescent state. In the lymphoid system, however, Lin (1973) showed only a slight difference in response between proliferating and non-proliferating cells. Using Chinese hamster cells in culture Ray *et al.*, (1973) found no difference in response between exponential and plateau phase cells.

None of these workers have obtained dose-response curves for HN2 having initial shoulders, a phenomenon shown clearly in our results for plateau phase cells. We are currently investigating the situation regarding split-dose exposure to HN2 in our plateau phase cells, in order to see whether the shoulder represents any ability to accumulate and repair sublethal damage.

*BCNU*

In their original investigation of the relative response of exponential and plateau phase Chinese hamster ovary cells to BCNU, Barranco, Novak and Humphrey (1973) found that exponential phase cells were very much less sensitive to this agent. This finding was supported by the observations of Hageman, Schenken and Lescher (1973) using a murine mastocytoma either *in vitro* or as an ascites tumour *in vivo*. However, Thatcher and Walker (1969) found no difference in sensitivity to BCNU as embryonic hamster cells move from exponential into stationary phase. More recently, Hahn, Gordon and Kurkjian (1974) have pointed out that response to BCNU *in vitro* is very dependent upon the serum content of the medium to which the drug is added. The results of Barranco *et al.* (1973*a*) and Hagemann *et al.* (1973) both of whom added BCNU directly to the existing medium in which the cells were growing, could therefore have been influenced by changes in drug binding properties between exponential and plateau phase medium. Nevertheless, Hahn *et al.* (1974) were able to show that plateau phase cells are somewhat more sensitive to BCNU than exponentially growing cells, even when exposed to the drug without serum being present.

Our results indicate little difference in the sensitivity of exponential, early and late plateau phase cells, even though we carried out the experiments by adding the drug directly to the growth medium. This method in the study of Hahn *et al.* (1974) greatly increased the differential sensitivity between exponential and plateau phase cells. Our experiments were carried out over a period of several months and using several different serum batches, and this could well explain the rather wide spread seen between the results of individual experiments. In no single experiment, however, did we find exponential phase cells to be considerably less sensitive than cells in plateau phase. The small differential between the exponential and early plateau phases seen in our early experiments (Twentyman and Bleehen, 1973), was not confirmed by these more complete studies.

In the bone marrow, BCNU has been found to have more effect against rapidly proliferating colony-forming units (van Putten, Lelieveld and Kram-Idsenga, 1972).

*CCNU*

Most of the remarks which we have made concerning BCNU also apply to CCNU. Barranco *et al.* (1975) have found this agent to have less effect against exponentially growing cells, even when exposure to the drug is carried out in a serum-free medium. In the bone marrow, van Putten *et al.* (1972) have again found more effect of CCNU against rapidly than against slowly proliferating colony forming units.

The results which we have presented in this paper are in several ways conflicting with results obtained in various other plateau phase systems. We do not claim that our results are in any way more correct than those of other workers, only that they represent what happens for one cell line under a very specific set of conditions.

It should also be pointed out that, in this type of study, the drug available per cell is greater in exponential phase cultures than in plateau phase for the same concentration expressed in $\mu$g/ml. This is intrinsic in the methodology and may be a factor in the results obtained. It does, however, apply to the results of the other workers as well as to our own results.

In our earlier report of the sensitivity of exponential, early plateau and late plateau phase cells to bleomycin (Twentyman and Bleehen, 1975), we analysed the results of various workers in different cell systems. We were unable to find any correlation between bleomycin sensitivity and either proliferative state or viability of the populations. It is evident that if plateau phase cells are to be useful as an *in vitro* model of tumour cells *in vivo*, much more must be learnt of the factors (*e.g.* membrane permeability, repair mechanisms) which determine the response to cytotoxic drugs of cells *in vitro*. We are presently studying some of these factors. On the other hand, it may be that there is no satisfactory way of representing *in vitro* the complex environmental situation existing *in vivo*. In any case, *in vitro* tests at our present stage of knowledge can act only as a very preliminary guide to possible responses which may or may not be confirmed by subsequent work using experimental tumour systems.

This work was partly financed by a grant from the Cancer Research Campaign which we gratefully acknowledge. BNCU and CCNU were kindly supplied by the Drug Development Branch, Division of Cancer Treatment of the United States National Cancer Institute. We thank Stella Keller and Keith Rowlinson for technical assistance.

### REFERENCES

BARRANCO, S. C., NOVAK, J. K. & HUMPHREY, R. M. (1973*a*) Response of Mammalian Cells following Treatment with Bleomycin and 1,3-Bis (2-chloroethyl)-1-nitrosourea during Plateau Phase. *Cancer Res.*, **33**, 691.

BARRANCO, S. C., GERNER, E. W., BURK, K. H. & HUMPHREY R. M. (1973*b*) Survival and Cell Kinetics Effects of Adriamycin on Mammalian Cells. *Cancer Res.*, **33**, 11.

BARRANCO, S. C., NOVAK, J. K. & HUMPHREY, R. M. (1975) Studies on Recovery from Chemically-induced Damage in Mammalian Cells. *Cancer Res.*, In the press

BARRANCO, S. C. & NOVAK, J. K. (1974) Survival Responses of Dividing and Non-dividing Mammalian Cells after Treatment with Hydroxyurea, Arabinosylcytosine or Adriamycin. *Cancer Res.*, **34**, 1616.

BIENKOWSKA, Z. M., DAWSON, K. B. & PEACOCK, J. H. (1973) Action of Actinomycin D, Bleomycin and X-rays on HeLa Cells.. *Br. J. Radiol.*, **46**, 619.

BLACKETT, N. M. & ADAMS, K. (1972) Cell Proliferation and the Action of Cytotoxic Agents on Haemopoietic Tissue. *Br. J. Haemat.* **23**, 751.,

BRUCE, W. R., MEEKER, B. E. & VALERIOTE, F. A. (1966) Comparison of the Sensitivity of Normal Hemopoietic and Transplanted Lymphoma Colony-forming Cells to Chemotherapeutic Agents Administered *in vivo*. *J. natn. Cancer Inst.*, **37**, 233.

DREWINKO, B. & GOTTLIEB, J. A. (1973) Survival Kinetics of Cultured Human Lymphoma Cells Exposed to Adriamycin. *Cancer Res.*, **33**, 1141.

ELKIND, M. M., SAKAMOTO, K. & KAMPER, C. (1968) Age-Dependent Toxic Properties of Actinomycin D and X-rays in Cultured Chinese Hamster Cells. *Cell Tissue Kinet.*, **1**, 209.

HAGEMANN, R. F., SCHENKEN, L. L. & LESHER, S. (1973) Tumor Chemotherapy: Efficacy Dependent on Mode of Growth. *J. natn. Cancer Inst.*, **50**, 467.

HAHN, G. M. & LITTLE, J. B. (1972) Plateau Phase Cultures of Mammalian Cells: An *in vitro* Model for Human Cancer. *Curr. top. Radiat. Res.*, **8**, 39.

HAHN, G. M., GORDON, L. F. & KURKJIAN, S. D. (1974) Responses of Cycling and Non-cycling Cells to 1,3-Bis (2-chloroethyl)-1- nitrosourea and to Bleomycin. *Cancer Res.*, **34**, 2373.

LIN, H. S. (1973) Differential Lethal Effect of Cytotoxic Agents on Proliferating and Non-proliferating Lymphoid Cells. *Cancer Res.*, **33**, 1716.

RAY, G. R., HAHN, G. M., BAGSHAW, M. A. & KURKJIAN, S. (1973) Cell Survival and Repair of Plateau-phase Cultures after Chemotherapy—Relevance to Tumor Therapy and to the *in vitro* Screening of New Agents. *Cancer Chemother. Rep.*, Pt 1, **57**, 473.

THATCHER, C. J., & WALKER, I. G. (1969) Sensitivity of Confluent and Cycling Embryonic Hamster Cells to Sulphur Mustard, 1-3 Bis(2-chloroethyl) -1-Nitrosourea and Actinomycin D. *J. natn. Cancer Inst.*, **42**, 363.

TWENTYMAN, P. R. & BLACKETT, N. M. (1970) Action of Cytotoxic Agents on the Erythroid System of the Mouse. *J. natn. Cancer Inst.*, **44**, 117.

TWENTYMAN, P. R. & BLEEHEN, N. M. (1973) The Sensitivity of Cells in Exponential and Stationary Phases of Growth to Bleomycin and to 1,3 Bis (2-chloroethyl)-1-nitrosurea. *Br. J. Cancer*, **28**, 500.

TWENTYMAN, P. R. & BLEEHEN, N. M. (1975) Changes in Sensitivity to Radiation and to Bleomycin Occuring during the Life-history of Monolayer Cultures of a Mouse Tumour Cell Line. *Br. J. Cancer*, **31**, 68.

TWENTYMAN, P. R., WATSON, J. V., BLEEHEN, N. M. & ROWLES, P. M. (1975) Changes in Cell Proliferation Kinetics Occuring During the Life-history of Monolayer Cultures of a Mouse Tumour Cell Line. *Cell Tissue Kinet.*, **8**, 41.

VAN PUTTEN, L. M. & LELIEVELD, P. (1971) Factors Determining Cell Killing by Chemotherapeutic Agents *in vivo*. II. Melphalan, Chlorambucil and Nitrogen Mustard. *Eur. J. Cancer*, **7**, 11.

VAN PUTTEN, L. M., LELIEVELD, P & KRAM-IDSENGA, L. K. J. (1972) Cell-cycle Specificity and Therapeutic Effectiveness of Cytotoxic Agents. *Cancer Chemother. Rep.*, **56**, 691.